# Effect of a traditional marinating on properties of rainbow trout fillet during chilled storage

**Published:** 2016-12-15

**Authors:** Siavash Maktabi, Mehdi Zarei, Milad Chadorbaf

**Affiliations:** 1 Department of Food Hygiene, Faculty of Veterinary Medicine, Shahid Chamran University of Ahvaz, Ahvaz, Iran; 2 DVM Graduate, Faculty of Veterinary Medicine, Shahid Chamran University of Ahvaz, Ahvaz, Iran

**Keywords:** Chemical changes, Marinating, microbial load, Rainbow trout, Refrigerator

## Abstract

In recent years, there has been an increasing interest in using food additives from natural sources to improve taste and also extend the shelf-life of semi-preserved foodstuffs. The aim of this study was to examine the chemical and microbiological changes promoted by a local marinating process in rainbow trout fillets during chilled storage. Fish fillets were immersed in marinades and stored at 4 ˚C for 10 days and were analyzed for total volatile basic nitrogen (TVN), thiobarbitoric acid (TBA), water holding capacity (WHC), pH, mesophilic and psychrophilic bacterial count every two days. Variations in TBA and WHC were not statistically significant between marinated and control groups. The values of TVN, pH, total psychrophilic bacteria count (TPC) and total mesophilic bacteria count (TMC) in marinated samples were significantly lower than controls. The most obvious finding of this study was that traditional marinated rainbow trout fillet stored in 4 ˚C had no undesirable changes at least for eight days.

## Introduction

The rainbow trout (*Oncorhynchus mykiss*) is one of the main fish species farmed in Iran. Rainbow trout is very closely related to salmon in containing the highest content of polyunsaturated fatty acids such as eicosapentaenoic acid and decosahexaenoic acid compared to other fish and seafood.^1^ The demand for rainbow trout in Iran has increased significantly over the last decade and this could be due to its desirable characteristics.^2^^-^^4^

Fatty fish such as rainbow trout have limited shelf-life and quality deterioration of this species is mainly caused by rapid growth of microorganisms and lipid oxidation.^5^ Thus, the off-odor and off-taste of the products affect the consumer acceptability.^6^^,^^7^ Today, the addition of synthetic preservatives, antioxidants, and colorants to extend the shelf-life of foodstuffs has been revised by authorities due to certain health problems. Scientists have become interested in using antioxidants from natural sources.^8^

Marinades including sugar, spices, oil, vinegar or fruit juice that already have been used to improve the tenderness, juiciness, flavor and aroma of foodstuffs, are the solutions.^9^ Marinating process slows down the bacterial and enzymatic activity and provides test tenderness, textural and structural changes with a prolonged shelf-life.^10^ To the best of our knowledge, several studies regarding seafood marinating by rosemary extract,^9^ tomato sauce,^11^ holy basil, paper-garlic,^12^ yogurt and spices^13^ and acetic acid with NaCl^14^ have been documented.

People in the south-west of Iran make a local marinade by immersion of fish fillets in a mixture of lemon juice, grated fresh garlic, salt, turmeric, red chili powder and black pepper. This marinade has a great reputation and the public interest as well. This method could be used as an industrial process in order to provide a semi-preserved and ready to cook fish for consumers using natural antioxidants. The aim of the present study was to investigate the chemical and microbiological changes promoted by the local marinating process in rainbow trout fillets during refrigerated storage.

## Materials and Methods

Raw materials and process. The study consisted of three trials and in every trial 40 fish fillet (approximately weighed 60 to 70 g) was used. Similar to a fish processing plant, the fillets were washed under tap water and placed in a clean basket to dry. Fillets were separated into two groups. The first group was immersed in a marinated mixture as described below for 3 hr at 4 ˚C. The second group was left in the same condition without marinating as the control.

Preparation of marinating mixture. In a bowel, 500 mL industrial lemon juice, 250 g grated fresh garlic, 60 g table salt, 3 g turmeric, 1 g hot chili powder, 3 g black pepper and 100 mL distilled water were mixed well as marinating mixture.

Sample preparation. All marinated and normal fish samples were separately placed in a plastic container with lid and incubated in a household refrigerator (4 ˚C) for 10 days and examined every two days for microbiological and chemical features. At the start of the experiments, three fish samples were randomly selected and aseptically chopped together thoroughly.

Chemical analysis. Total volatile basic nitrogen (TVN) were measured according to method of Malle and Poumeyrol.^15^ Briefly a 10 g fish flesh, 1 g magnesium oxide (Merck, Darmstadt, Germany) and 60 mL distilled water were placed in distilling flask. Samples were boiled and distilled into 40 mL of boric acid (Merck) containing methyl red as indicator. After the distillation, the contents of conical flask were titrated with H_2_So_4 _(Merck) and TVN was expressed as mg of N per 100 g muscle. The value of thiobarbituric acid (TBA) was determined according to Wrolstad et al.^16^ A portion of sample (5 g) was blended with 100 mL of 10% trichloracetic acid (Merck) for 3 min and filtered. The solution content was increased to 100 mL by adding extra trichloracetic acid (10%). Then, a 4 mL aliquot was transferred into test tubes and 5 mL thiobarbituric acid (0/02 w/w) was added and incubated at 90 ˚C for 1 hr. Absorbance was measured at 532 nm using a spectrophotometer (Model CE2040; Cecil, Cambridge, UK). The results expressed as mg MDA per kg muscle. The pH value was determined for the homogeneous mixtures of fish with distilled water (1:10, w/v), using a digital pH meter (Model PB-11; Sartorius AG, Goettingen, Germany) as described by benjakulet et al.^17^ Water holding capacity (WHC) was measured according to the method of Omana et al.^18^ Briefly, the weight of three disk of filter paper was measured and 0.3 g of fish fillet was placed on to them. A mass of 1 kg was applied for 2 min. The weight of filter paper was measured again and the percentage of WHC was calculated by relevant equation.

Microbiological analysis. For enumeration of bacteria, 10 g of the fish muscle was homogenized in 90 mL of normal saline and stomached for 3 min. Further decimal dilutions were made, and then 0.1 mL of each dilution was streaked on the surface of nutrient agar (Fluka 70152; Steinheim, Switzerland) plates. Two series of cultured agar were made and the first group was incubated for 7 days at 7.0 ± 0.5 ˚C for estimation of total psychrophilic bacteria count (TPC). The second group was incubated for 24 hr at 35 ˚C for total mesophilic bacteria count (TMC).

Statistical analysis. All experiments were performed in triplicate. Results were analyzed by repeated measure analysis of variance (ANOVA) using SPSS (version 16; SPSS Inc., Chicago, USA). The significance levels are expressed at 95% confidence level (p < 0.05) throughout.

## Results

**Fig. 1:**
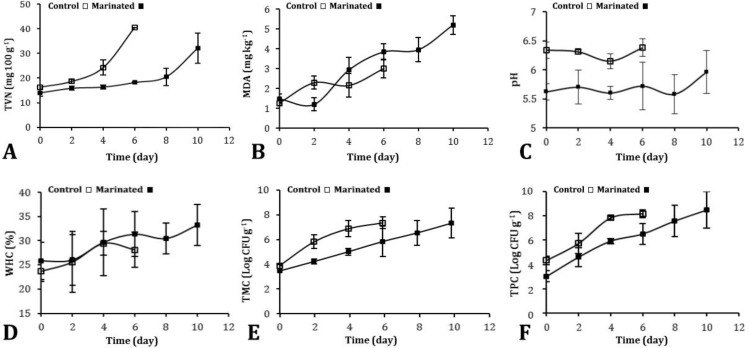
Means values and standard error of three (n=3) independent determinations for value of A) Total volatile basic nitrogen (TVN); B) Thiobarbituric acid (TBA); C) pH; C) pH; D) Water holding capacity (WHC);  E) Total mesophilic bacteria count (TMC) and F) Total psychrophilic bacteria count (TPC) in control and marinated fish samples

 The TVN value for control and marinated samples are presented in Fig. 1A. The initial value for marinated samples (15 mg per 100 g) was increased during storage. This amount on day six was 17 and reached 32 mg per 100 g at the end of storage. The value of control samples dramatically increased. This amount reached 40 mg per 100 g on day 6. After that, due to sample putrefaction, further investigation was impossible.

Statistical analysis showed a significant difference in TVN values (p < 0.05) between control and marinated samples. This indicates that marinade cause delayed protein degradation and spoilage of fish.

Figure 1B shows the changes in TBA value (mg malonaldehyde per kg) from days 0 to 10 for control and marinated samples. The initial TBA value in both groups was about 1.4 mg kg^-1^ and increased in parallel during storage period. This amount on day six was 3.0 and 3.8 mg kg^-1^ for control and marinated sample, respectively. The highest recorded value was 5.2 mg kg^-1^ and for marinated samples at the end of storage. Statistical analysis shows that the difference in TBA values between both groups was not significant (p > 0.05).

Initial pH of marinated and control fish samples was 5.3 and 6.7, respectively (Fig. 1C). This value for both groups was almost stable after 10 days of storage. A significant difference (p < 0.05) between control and marinated groups was observed in statistical analysis. This difference could be due to use of acetic acid (lemon juice) for fish marinating.

The changes in WHC value are shown in Fig. 1D. Although this amount was increased for both groups during storage, the differences between marinated and control samples was not significant.

As shown in Fig. 1E, the number of mesophilic bacterial in marinated samples increased from 3.7 log (cfu per g) on the first day to 5.5 log on day 6; while in the control group, the amount was increased from 3.9 to 7.1 log which was almost 1.5 log higher. Finally, in marinated samples the value increased to 7.3 log at the end of storage period. The number of psychrophilic bacteria in control samples 3.5 log increased after six days of storage, while the amount for marinated samples was 2.3 log (Fig. 1F). The results indicated a positive effect of marinade in control of bacterial growth.

## Discussion

 The value of TVN is a good indicator for determining the spoilage levels in fresh and semi-preserved seafood.^1^ The initial level of TVN between 10.0 to 21.0 mg per 100 g in fresh rainbow trout flesh has been estimated.^19^^,^^20^ Also, the highest acceptable level of TVN in rainbow trout fillets stored in modified atmosphere is 25,000 mg per 100 g.^12^ In seafood, the maximum acceptable level of TVN is 35.0 mg per 100 g.^21^^,^^22^ In a study by Pakawatchaiet al.,the effects of the antioxidant and antibacterial activities of herb and spice pastes, holy basil and pepper-garlic, on minced salmon flesh waste stored at 4 ˚C was investigated.^12^ They reported that the initial level of TVN in all samples was 9 mg kg^-1^ and increased to 12.0 to 14.0 mg per 100 g over 12 days storage. Surprisingly, the control sample had the lowest TVN value during storage. However, the findings of this study do not support the previous research that the level of TVN in marinated samples was lower than controls.^14^^,^^23^

In the present study, The TVN level in control samples was starting to increase from day two and reached 40.0 mg per 100 g on the 6^th^ day of storage which was an un-acceptable level. Organoleptic findings confirmed this result because of smell of corruption and putrefaction (data not reported). However, the TVN level in marinated samples was still under acceptable level (less than 35 mg per 100 g) after 10 days of storage. This may indicate that the marinade, delays protein degradation and fish flesh putrefaction.

The analysis of TBA is widely used for the detection of oxidative rancidity in oil and food samples.^24^ In some studies, the maximum TBA value in a good quality of frozen fish, chilled or stored with ice, 5.0 mg malon-aldehyde per kg and for consumable fish flesh, up to level of 8 mg malonaldehyde kg^-1^ has been reported.^22^ It seems that this value may be variable based on fish type, temperature and storage time. For example, 15 mg malonaldehyde kg^-1^ has also been proposed as the acceptable TBA value in mackerel fish stored in – 3 to 10 ˚C.^25^ This value for rainbow trout fillet after 6 and 12 days stored in 4 ˚C has been recommended to be 1.0 and 1.8 mg kg^-1^, respectively, which is much less than mackerel fish.^26^ In another study, a TBA value of up to 2.3 mg kg^-1^ has been calculated for vacuum packed rainbow trout fillet after 15 days storage in 4 ˚C. ^27^

The effect of fish marinating on changes of TBA value during storage has been the subject of few studies. For example, effects of combining of smoking and marinating by alcohol vinegar and salt on the shelf-life of anchovy stored at 4 ˚C was subject of an investigation. The value of TBA significantly increased from 1.9 to 4.2 mg kg^-1^ after six months storage.^20^ In another study the shelf-life of pasteurized and not-pasteurized sardine marinades with acetic acid, sodium chloride, tomato sauce and spices was investigated. The TBA value of pasteurized sardine increased from 4.3 mg kg^-1^ to 8.1 mg kg^-1^ and TBA value of not-pasteurized sardine increased from 4.4 mg kg^-1^ to 8.2 mg kg^-1^ at six months of storage. The differences between TBA and pH value of both groups were not significant.^11^

Rancidity in minced salmon flesh waste stored at 4 ˚C was investigated and it was shown that in sample with holy basil paste added, TBA value significantly was higher than the control. The sample added with pepper-garlic paste, TBA value was lower compared with the control after 12 day of storage. They concluded that this might be markedly due to the pro-oxidant effect of chlorophyll and other impurities existing in holy basil. Also, presence of antioxidant compounds such as alliin, diallyl sulphide, allyl sulphide and propyl sulphide derived from garlic and piperine in black pepper could retard lipid oxidation immediately during mixing and during storage.^12^ An antioxidant effect of turmeric was observed in vacuum packed rainbow trout fillet during a15-day of storage at 4 ˚C. The TBA value in marinated samples was double than the control after 10 days storage.^27^ Again, this result was attributed to the antioxidant effect of turmeric. In another study, the TBA value on rainbow trout stored at 4 ˚C from 0.1 mg kg^-1^ on first day increased to 2 mg kg^-1^ on day 12 and then reduced to 1.4 on day 18.^26^

In our investigation, the TBA value was calculated until the 6^th^ day of storage due to sample purification. Our data showed that the TBA value for both control and marinated samples increased in parallel during storage. Also, as shown in Fig. 1B, in marinated samples the value decreased until the second day and then rose again. These findings were in agreement with previous studies which mentioned that the TBA value may reduce several days after storage.^28^^,^^29^ In contrast with previous researches^26^^,^^27^ The TBA value for both groups was slightly high. However, the differences between the TBA value in marinated and control samples was not significant and it seemed that this factor alone could not be a reliable parameter to determine fish spoilage during chilled storage.

Our marinade contains several ingredients that some like black pepper and turmeric have antioxidant effect and some like crushed garlic extract may induce oxidation in lipids and an overlapping effect could happen.^30^ It must be mentioned that despite of high TBA value, sensory finding on day eight did not show a major undesirable changes in marinated samples so the marinades might be helpful to postpone spoilage signs.

The pH changes could be useful to evaluate the qualitative changes in fish during storage. The initial pH in fresh fish flesh is in range of neutral but decomposition of nitrogenous compounds leads to an increase in pH during storage.^31^ The increase in pH indicates the loss of quality. In contrast, decrease of pH in stored fish flesh could be observed due to the acid which is a common metabolite from growth of a number of bacteria include lactic acid bacteria, Enterobacteriace and Photobacterium phosphorum.^32^ The initial pH value in different fish are: anchovy 5.8,^20^ rainbow trout 6.7,^27^ andsardine 5.8 to 6.2.^33^^-^^35^ In the present study, the initial pH value of the marinated samples (5.3) was significantly lower than control samples (6.7) and was almost stable in both groups during storage. A similar result to our findings was observed by Özogul et al., where pH was stable in smoked and marinades anchovy after six month storage.^20^ However, the findings of the current study do not support other researches. For example, pH decreased in marinades rainbow trout in turmeric extract after 20 days storage at 4 ˚C,^27^ or increased in sardine marinades in tomato sauce according to storage time.^11^

As mentioned before, significant difference in WHC value between marinated and control samples were not observed. Various factors, such as the addition of acid, temperature changes, enzymatic and bacterial degradation can be effective in this regard.

Growth of bacteria is one of the most important factors in spoilage of fish. The mean of initial number of bacteria in our samples was between 3.0 to 4.0 log CFU per g, indicating that the fish used in this study had good quality.^36^^-^^39^ Also in this study, an initial reduction in bacterial viable counts was observed by marinades. The present findings seem to be consistent with other research which found that microbial load decreased just after the shrimps were marinated.^9^

As it showed in Figs. 1E and 1F marinating process was effective on psychrophilic and mesophilic bacteria and TVC in marinated samples was significantly lower than controls. Also, it has been reported that bacteria are not completely killed by marinating and live cells are still able to grow in marinated samples. In this condition, they are able to continue their activity more or less rapidly according to their ability to adapt to the medium during storage.^40^ This also accords with previous observations, which showed a positive effect on reducing the number of bacteria in the samples stored at different conditions using natural preservatives such as mixture of garlic and black pepper,^12^ turmeric, ^27^ tomato sauce^11^ and mixture of acetic acid and salt.^14^

In conclusion, this investigation showed that marinating of rainbow trout fillets by a special local marinade enhanced the shelf-life at 4 ˚C by preventing protein degradation and delay microbial spoilage. The results of microbiological and chemical evaluation indicated that marinated fillets maintained their good quality characteristics at least two days longer than control samples. With this method, flavored ready to eat fish fillet could be available for consumers with longer shelf-life.
